# The World Health Organization targets for cervical cancer control by 2030: a baseline assessment in six African countries—part I

**DOI:** 10.3332/ecancer.2022.1453

**Published:** 2022-10-07

**Authors:** Daniela Cristina Stefan, Jean-Marie Dangou, Prebo Barango, Issimouha Dille Mahamadou, Sharon Kapambwe

**Affiliations:** 1 Institute of Global Health Equity Research, University of Global Health Equity, 0000 Kigali, Rwanda; 2 World Health Organization Regional Office for Africa, P.O. Box: 06, Brazzaville, Republic of the Congo

**Keywords:** cancer control, cervical cancer, WHO targets 2030, cancer care personnel, pathologists, personnel training

## Abstract

**Aim:**

We present and analyse the findings of a survey of the readiness of the healthcare systems in Eswatini, Guinea, Malawi, Rwanda, Uganda and Zambia, to implement the necessary measures for attaining the targets for cervical cancer control, set by The World Health Organization (WHO), by the year 2030.

**Methods:**

A questionnaire with 129 questions with preset answer options was completed in 2020, by ministries of health programme coordinators for non-communicable diseases, cancer control and/or reproductive health, and by WHO country offices, in the six countries selected.

**Results:**

The findings on demographics, burden of disease, governance and management, laboratory services, equipment, supplies and medicines, as well as on personnel and training are presented here. The burden of cervical cancer in the countries studied is considerable, according to The International Agency for Research on Cancer estimations. The incidence of the disease is augmented by the high prevalence of HIV infection, in most of the countries surveyed. Most of the population live in rural areas, where access to the health services is far from ideal. Facilities for screening with human papilloma virus tests and for histopathology are limited. One pathologist covers the diagnostic needs of between 0.5 and 4 million inhabitants. Most other categories of health professionals are under-represented, and the capacity to train them is inadequate.

**Conclusions:**

Strong country commitment and leadership, innovative solutions and extensive international cooperation would be needed to attain the targets of cervical cancer control set by WHO, in these countries.

## Introduction

The International Agency for Research on Cancer (IARC) estimated that in 2020, close to 342,000 women died from cancer of the uterine cervix, in the world. The age-standardised mortality from the disease was 17.4/100,000 in low-income populations, seven times higher than the 2.5/100,000 recorded in the high-income populations. In the same year, according to IARC, around 603,300 new cases of cervical cancer appeared worldwide [1].

According to IARC predictions, this figure will grow to 800,000 by 2040. A large share of these new cases will appear in low- and low middle income countries. The African continent would see its number of incident cases almost double in the next 20 years, from 117,000 to 228,000 [2].

By implementing adequate measures for cervical cancer control, this ascending trend can be reversed. It is already possible to prevent cervical cancer by vaccinating young girls against the human papilloma virus (HPV), before the onset of sexual activity [3], and by screening women for precancerous changes, to remove those, if diagnosed. Cervical cancers detected and treated in early invasive stages have high rates of cure [4]. The difficulties on the way to implementing the measures mentioned are substantial, but can be overcome. The reward would be that sometime in the future, cancer of the cervix would be relegated to the history of medicine.

In 2018, the Director-General of The World Health Organization (WHO) made a call to action for elimination of cervical cancer as a public health problem [5]. To achieve this aim, the world-wide incidence of the disease should be brought below 4/100,000 women. A global strategy towards that goal was adopted by the World Health Assembly in 2020. First targets, to be attained by 2030, consist of: 1) world-wide vaccination of 90% of girls by 15 years of age, 2) screening of 70% of women with a high-performance test for detection of pre-cancerous lesions of the cervix, by the age of 35 and again by the age of 45 and 3) treating 90% of the detected pre-invasive cancers and managing 90% of women with invasive cancers [6].

The Non-Communicable Disease division of WHO/AFRO (Regional Office for Africa) selected to support a ‘first wave’ of six countries, in their effort to achieve the above targets: Eswatini, Guinea, Malawi, Rwanda, Uganda and Zambia. The experience gained in the process will be used to extend the support to other countries on the continent.

We report the findings of an evaluation by AFRO, of the readiness to implement and to scale up the measures necessary for the achievement of the targets set for 2030, in the first wave countries. This, the first part of the report, presents findings concerning demographics, burden of disease, governance and management, laboratory services, equipment, supplies and medicines, as well as personnel and training will be presented. The remaining data, including the state of primary and secondary prevention, of the treatment with curative intent and the state of palliation, will be published in a separate report.

## Methods

A detailed questionnaire in English was mailed in May 2020 to national programme coordinators for non-communicable diseases, cancer control and/or reproductive health national programme coordinators, Ministries of Health, representatives and WHO country offices. The officials collaborated with each other in drawing the answers, resulting in a single completed questionnaire for each country.

The 129 questions provided preset answer options. Further consultations were held via internet for omitted questions or answers requiring clarification. Consultations were held in English, except for Guinea representative which were held in French. The results were compiled in comparative tables (Tables 1–8) as shown below.

## Results

### Demographics and epidemiology

Excepting Zambia, in the countries surveyed, from 64% to 84% of the female population aged between 35 and 49 years live in rural areas. The numbers of HIV infected females vary within a broad range, from 1.5% in Guinea to 32.5% in Eswatini. The female literacy rate varied from 22% in Guinea to 95.6% in Eswatini.

Excepting Uganda and Rwanda, the age-standardised incidence of cervical cancer in the countries surveyed is two to three times higher than the average figure for Africa, which is 25.6/100,000/year. The mortality figures, excluding Rwanda, are also twice or even three times higher than the average of 17.7/100,000/year for the whole continent [9].

### Governance and management

Most of the first wave countries have national cancer control plans, as well as cervical cancer control plans and guidelines, drawn according to WHO guidelines. However, although provisions for monitoring and evaluation of the activities planned are made, a specific team tasked with monitoring and evaluation exists only in the Rwandan Department of Health.

### Laboratories

The above data, correlated with the respective countries’ population, indicate a ratio of 1 pathologist for between 0.5 and 4 million inhabitants, depending on country.

### Equipment, supplies and medicines

Cryotherapy, thermal ablation and LLETZ equipment is available in most countries surveyed; there is no LLETZ in Uganda and no thermal ablation in Eswatini. Opiates are not available in Guinea.

### Personnel and training

Only Uganda and Zambia have programmes for training gynaecological oncologists. Pathologists, crucial for the diagnosis and management plan of cancers, are only being trained in Rwanda, Uganda and Zambia.

Zambia estimates the highest need of professionals in almost all fields related to oncology. Malawi did not provide an answer to this question. Uganda did not report a definite need for some of the professions.

## Discussion

### Cancer registration

The first wave countries cumulatively have a female population of almost 55,200,000. In 2020, it is estimated that 17,903 women were diagnosed with cervical cancer there and 11,922 women died. It is important to be aware that these reported estimations are provided by IARC and not by country cervical cancer registries. Data on invasive cervical cancer, screening for pre-invasive lesions or on vaccination are not centralised. For realistic planning by the Health Ministries, including an efficient allocation of resources, reliable national cervical cancer data registration should be available.

### Governance and management

Most first wave countries report having national cancer control plans, except for Guinea. Apart from Uganda and Guinea, all countries have cervical cancer control plans and guidelines. However, there is a clear need to institute a mechanism for evaluation of the results of those plans and guidelines in practice. Of the six respondents, only Rwanda had a cervical cancer control monitoring team at governmental level.

It is remarkable that four of the countries surveyed have governmental measures in place to develop e-Health facilities; they have e-Health units as well. The role of eHealth systems is to deliver health services and information through the internet. Medical tele-communication may prove a determinant factor for the success of fighting cervical cancer: it will make it easier to centralise and assess data on the disease burden, to organise doctor-to-doctor consultations on difficult cases, as well as online tumour boards, to access scientific information for clinical decision support and to consult guidelines. As it will be shown in the next section, the telepathology facility will be indispensable for the diagnosis and management of cervical cancer in Africa.

As part of the e-Health activities, the m-Health (via mobile telephone) could be leveraged to deliver information on the disease, as well as on the HPV vaccination and screening. It may also be used for maintaining the patients in the system, by means of appointments, reminders or navigation of the referral pathways, for the whole duration of their management.

### Laboratories and pathologists

The ratios of laboratories and pathologists to population in the first wave countries are not reassuring. The figures for pathologists are far behind the best ratios in Africa, of between 1/50,000 and 1/200,000 inhabitants [10], registered in Algeria and Tunisia. In UK, where the density of pathologists is 1 for little under 30,000 inhabitants, a survey published in 2018 by The Royal College of Pathologists [11] found that 78% of the departments surveyed needed more consultants. Moreover, pathologists do not work alone; their task is facilitated by medical technologists or technicians, histo- and cyto-technologists, information technology specialists, forensic pathology technicians, administrative and clerical personnel and others. It can easily be inferred that the pathology workforce in the countries surveyed would require considerable reinforcement.

The cervical cancer screening targets set for WHO for 2030 require large-scale shifting from inspection of the cervix with acetic acid or Pap smear to HPV testing. This testing is automatised; however, besides acquiring the machines capable of performing the test, suitable infrastructure, including a reliable provision of clean water, electricity and distance communication, as well as personnel trained to prepare the samples for analysis, service the machine and attend to possible breakdowns, should be available. Additionally, at present, the HPV testing circuit are not well suited for a test-and-treat approach, hence the need for an efficient follow-up system for women identified as high-risk.

Further, the scaling up of screening would generate considerably more cytology smears and cervical biopsies, hence a substantial increase in demand for cytopathological and histopathological diagnoses. With the 18 full pathology courses offered in Africa at the present, it was calculated that only in several centuries will Sub-Saharan Africa attain the present ratios of pathologists to population found in US or UK [10]. Besides training personnel abroad, the acquisition of slide scanners with connectivity to the Internet should be imperiously considered. This would make possible the outsourcing of slide reading, either to human pathologists or to artificial intelligence systems (AI), located at distance, even beyond borders. For image evaluation, AI is as performant as humans or even better, to the point where in a not too far future it may be considered unethical not to use it when examining a slide [12]^.^

### Equipment, supplies and medicines

Four of the surveyed countries had frequent shortages of chemotherapy agents for the cervical cancer. Additionally, Guinea reported a lack of opiates. An in-depth analysis of the causes could not be conducted at the time of the survey.

Another significant finding is that brachytherapy is available only in Zambia and Uganda. Eswatini and Malawi reported no radiotherapy facilities for cervical cancer. Given that most patients contact the health institutions late in the course of the disease, when surgery is not indicated, and radiation or chemo-radiation are the indicated therapies, many women might not have access to proper treatment in these countries. To attain the 90% rate of treatment envisaged by WHO by 2030, it would be necessary to build more radiotherapy facilities, including brachytherapy. Radiotherapy is used for other cancers too: calculations estimate that the present deficit of radiation centres in Africa is over 400 units [13]. The initial costs for a radiation bunker and brachytherapy machine, including the radioactive sources for the first year would amount to around 1.3 million US dollars [14], while a new linear accelerator may cost up to 2.8 million dollars.

### Personnel and training

Our data show a general shortage of personnel, in all professions related to the prevention and treatment of cervical cancer, in the countries studied. It is perhaps useful to highlight the overall need for 191 radiologists, 537 anaesthesiologists, 663 radiographers, 2,688 laboratory technologists, as more salient figures. And these figures are only for the six countries surveyed.

These needs reflect the present situation and do not include the personnel needed to accomplish the objectives set for 2030. The complexity of planning the healthcare human resources needed in the future was described, without giving definitive solutions, repeatedly in the literature [15–17]. However daunting that task may be, it needs to be accomplished in the countries studied, as a first step towards attaining the targets set by WHO for 2030 [18].

Training the doctors, nurses and other medical personnel mentioned in Table 8 will probably not be possible inside the countries studied, as some do not have any training facilities. Some of the people aspiring to enter these professions might be prepared to undergo their training abroad. Therefore, the existing agreements with various academic institutions in other countries or continents need to be revised and updated while new agreements may need to be entered, to cover as much as possible the needs. This activity should take place within the frame of a ministerial human resources for health strategic plan.

At the same time, the governments of the countries surveyed may realise, based on a thorough analysis of personnel needs, that the opening or upgrading of national institutions of training in health professions is long overdue and needs to happen. However, given the long duration of training of specialists in gynaecological oncological surgery, radiotherapy, pathology, anaesthesia and intensive care – to mention just a few – interim solutions, aimed at the 2030 targets, might need to be found.

Further, agreements could be concluded with medical schools from other African countries or even from overseas countries, to organise supervised training stages for their Master students, in the countries we surveyed. All the same, young specialists from foreign countries, particularly from Europe, may be recruited and accredited to work for limited periods, in areas of need. The benefit would be mutual: young specialists would be exposed to developing countries’ health and healthcare characteristics and gain valuable experience, while the population would benefit from improved prevention and treatment of cervical cancer.

### What is new in this report?

This is an analysis of a survey aimed at evaluating the readiness of six African countries to achieve the targets formulated by WHO regarding the reduction of the burden of cervical cancer by 2030. The respondents were high authorities from national health administrations and WHO country representatives, thus ensuring the veracity of the data published here.

### What are the shortcomings of this study?

The data collated here were only as good as the primary sources. The most obvious example is that in the absence of population-based cancer registries and well-coordinated national cancer (or cervical cancer) registries, the figures for incidence and mortality reported were those estimated by IARC. Another shortcoming was that some questions were not answered.

## Conclusions

The burden of cervical cancer in the countries studied is considerable, based on the IARC estimations. The incidence of the disease is augmented by the high prevalence of HIV infection, in most of the countries surveyed. Most of the population lives in rural areas, where access to the health services is far from ideal. Facilities for screening with HPV DNA tests are severely limited, and histopathology services are insufficient. One pathologist is expected to cover the diagnostic needs of between 0.5 and 4 million inhabitants. Most other categories of health professionals are under-represented or absent. Strong country commitment and leadership, innovative solutions and extensive sub-regional and international cooperation would be rather urgently needed to attain the targets of cervical cancer control set by WHO, in these countries.

## Funding

None.

## Conflicts of interest

None declared.

## Figures and Tables

**Table 1.** Demographics.

Country	Total population	Total female population	35–49 years	HIV females all ages (%)	WLHIV^a^ Urban versus rural (%)	Literacy rate – female (%)	Urban/rural females 35–49 years (%)
Eswatini	1,093,238	562,127	80,890	32.5	27.1/72.9	95.6	33/67
Guinea	12,559,624	6,447,777	840,963	1.5	2.4/1.2	22	36/64
Malawi	17,563,749	9,440,000	1,791,502	12.5	17.8/8.5	65.9	16/84
Rwanda	12,663,116	6,508,671	1,207,597	2.2	4.8/2.5	80	16/84
Uganda	45,740.000^b^	23,190,000^b^	2,529,000	7.6	N/A	61.97	20/80
Zambia	17,885,422	9,033,248	1,139,437	14.2	64/36	66	43/57

a Women living with HIV

b Statista 2021 [7].

**Table 2.** Burden of disease and registries available.

Cancer Today (ASR^a^ – World) in 2020			Country registries			
Country	Incidence of invasive cervix cancer (/100,000 per year)	Mortality due to cervix cancer (/100,000 per year)	Cancer registry	Registry of invasive cervical cancer	Registry of screening and precancerous lesions	Registry of immunised for HPV
Eswatini	84.5	55.7	Yes	No	At units	No
Guinea	50.1	37.2	Yes	No	At hospital	No
Malawi	67.9	51.5	Yes	No	At national level	Yes
Rwanda	28.2	20.1	Yes	No	At units	No
Uganda	13.3	41.4	Yes (Kyadondo district)	No	Yes	Yes
Zambia	66.4^b^	44.5^b^	Yes (Lusaka district)	At units	At units	No

a ASR, Age-standardised rate

b Data from 2018 [8]

**Table 3.** Governance and management. Cervical cancer prevention and control programmes.

Country	Health policy, plan, strategy	MOH^d^ cervical cancer existing unit	National Cancer Control Plan	National policy, plan or strategy eHealth^a^	Unit e-Health	HPV vaccination programme/unit	Cervical cancer policy, plan or strategy	Guidelines	National M&E^b^ plan CCNP^c^	MOHsection, unit or team M&E
Eswatini	Yes	Yes	Yes	No	No	Yes	Yes	Yes	Yes	No
Guinea	No	Yes	No	Yes	Yes	No	No	Yes	Yes	No
Malawi	Yes	Yes	Yes	Yes	Yes	Yes	Yes	Yes	Yes	N/A
Rwanda	Yes	Yes	Yes	Yes	Yes	Yes	Yes	Yes	Yes	Yes
Uganda	Yes	Yes	Yes	Yes	Yes	Yes	No	No	No	No
Zambia	Yes	Yes	Yes	N/A	N/A	Yes	Yes	Yes	N/A	N/A

N/A, Data not available

a eHealth, health services and information delivered through the internet

b M&E, Monitoring and evaluation

c CCNP, Cervical Cancer National Plan

d MOH, Ministry Of Health

**Table 4.** Laboratories and pathologists.

Country	National policy, plan or strategy on laboratories	Laboratories accreditation plan	Laboratory system centralised	National reference laboratory	Public		Private	
					Laboratories	Pathologists	Laboratories	Pathologists
Eswatini	No	No	Yes	Yes	1	1	2	1
Guinea	No	No	No	Yes	2	3	0	0
Malawi	Yes	Yes	Yes	Yes	4	4 (incl. private)	2	4 (incl. public)
Rwanda	Yes	Yes	Yes	Yes	5	15 (incl. private)	1	15 (incl. public)
Uganda	Yes	Yes	Yes	Yes	4	5	10	10
Zambia	Yes	No	Yes^a^	Yes	5	15 (incl. private)	5	15 (incl. public)

a At regional level

**Table 5.** State of HPV testing and histology.

Country	Providing HPV testing			HPV results recorded	Challenges to cervical cancer histology
	Public	Private	NGO		
Eswatini	0	0	0	0	N/A
Guinea	0	0	0	0	N/A
Malawi	0	1	2	N/A	Under research (data not available)
Rwanda	14	0	0	No	Lacking machinery and consumables
Uganda	11	2	1	No	N/A
Zambia	0	1	1	No	Old histopathology equipment frequently breaking down; poor supply of consumables

N/A, Data not available

**Table 6.** Challenges to the treatment of precancerous lesions; equipment and medicines availability.

Country	Challenge to treatment of precancerous lesions	Chemo agents on the essential list	Chemo for treatment of cervical cancer	Opiate pain medication availability	List of essential medical devices	Quality of medicines	National blood transfusion service
Eswatini	Liquid nitrogen, LLETZ^a^ loops	Yes	Frequent stockout	Infrequent stockout	No	Yes	Yes
Guinea	Liquid nitrogen, loops LLETZ	Yes	No	No	No	No	Yes
Malawi	Thermal ablation, LLETZ machines	Yes	Frequent stockout	Frequent stockout	Yes	Yes	Yes
Rwanda	Liquid nitrogen, LLETZ loops	Yes	Yes	Yes	No	Yes	Yes
Uganda	Cryotherapy, thermal ablation, LLETZ machines	Yes	Frequent stockout	Yes	Yes	Yes	Yes
Zambia	Machines for thermal ablation and LLETZ	Yes	Yes	Yes	Yes	Yes	Yes

a LLETZ, Large Loop Excision of the Transformation Zone

**Table 7.** Radiodiagnostic and radiotherapy machines.

Country	Radiodiagnostic			Radiotherapy		
	Computed tomography (total/functional)	Magnetic resonance imaging (total/functional)	Positron emission tomography	Linear accelerator	Telecobalt	Brachy-therapy
Eswatini	2/0	1	0	0	0	0
Guinea	5/3	2/1	0	0	0	0
Malawi	3/3	1/0	0	0	0	0
Rwanda	10/9	2/2	0	2/2	0	0
Uganda	10/7	3/1	0	1	2	1
Zambia	16	3	0	1	2	2

**Table 8.** Training possibilities and estimated need of professionals in the field of cancer care.

Profession		Eswatini	Guinea	Malawi	Rwanda	Uganda	Zambia
Existing accredited training	Clinical oncology	No	No	No	No	No	Yes
	Surgical oncology	No	No	No	No	No	No
	Gynaecologic oncology	No	No	No	No	Yes	Yes
	Urologic oncology	No	No	No	No	No	No
	Haematology	No	No	No	No	Yes	No
	Paediatric oncology haematology	No	No	No	No	Yes	No
	Palliative care	No	No	No	No	Yes	No
	Pathology	No	No	No	Yes	Yes	Yes
	Brachytherapy	No	No	No	No	No	No
	Other/specify	No	No	No	Nursing oncology	No	No
	Lab. scientist/technologist	4	NO	N/A^a^	1,256	N/A	1,428
	Needed	4	NO	N/A	574	N/A	2,110
	Pathologist	1	3	N/A	15	24	15
	Needed	2	15	N/A	10	N/A	70
	Pathology technologist	2	3	N/A	0	N/A	0
	Needed	7	30	N/A	30	N/A	280
	Radiographer/technologist	8	6	N/A	200	8	419
	Needed	10	76	N/A	15	20	542
	Radiologist	0	6	N/A	10	N/A	8
Health workforce numbers	Needed	1	76	N/A	47	N/A	70
	Nuclear medicine specialist	0	0	N/A	0	2	3
	Needed	2	8	N/A	5	N/A	70
	Endoscopist/gastroenterologist	2	4	N/A	0	5	5
	Needed	?	14	N/A	10	N/A	70
	Anaesthesiologist	4	10	N/A	22	12	25
	Needed	4	30	N/A	43	N/A	460
	Surgical oncologist	0	2	N/A	0	1	1
	Needed	1	15	N/A	10	22	32
	Gynaecologic oncologist	0	2	N/A	0	6	2
	Needed	2	20	N/A	5	22	32
	Urologic oncologist	0	5	N/A	0	0	0
	Needed	2	10	N/A	5	22	32
	Medical physicist	0	0	N/A	1	5	5
	Needed	2	16	N/A	4	10	8
	Radiation therapy technician	0	0	N/A	0	9	25
	Needed	2	16	N/A	15	20	75
	Clinical oncologist	1	1	N/A	5	3	7
	Needed	2	10	N/A	10	7	30
	Palliative care specialist	1	0	N/A	5	5	3
	Needed	10	15	N/A	20	32	116
	Oncology nurses	2	0	N/A	9	3	12
	Needed	10	30	N/A	50	40	30
	Nutritionist	4	0	N/A	200	N/A	5
	Needed	10	10	N/A	360	N/A	20

a N/A, Not answered
